# Predictive Value of Naples Prognostic Score in Determining the Prognosis of Acute Pancreatitis—A Single-Center Experience

**DOI:** 10.3390/jcm15176659

**Published:** 2026-08-28

**Authors:** Fatih Sargin, Zeynep Gok Sargin, Ahmet Caliskan, Ayse Karaduru Avci, Celal Dogan, Ali Avci

**Affiliations:** 1 Department of Intensive Care, Antalya City Hospital, Antalya 07080, Turkey; sarginfatih@gmail.com; 2 Department of Gastroenterology and Hepatology, Antalya City Hospital, Antalya 07080, Turkey; 3 Department of Internal Medicine, Antalya City Hospital, Antalya 07080, Turkey; ahmetcaliskan0007@gmail.com (A.C.); aysekaraduru07@gmail.com (A.K.A.); 4 Department of Pulmonary Medicine, Antalya City Hospital, Antalya 07080, Turkey; dr.celaldogan@gmail.com; 5 Department of Emergency Medicine, Antalya City Hospital, Antalya 07080, Turkey; aliavci171@hotmail.com

**Keywords:** Naples prognostic score, acute pancreatitis, severity prediction, prognostic marker, severe acute pancreatitis

## Abstract

**Background/Objectives:** The Naples Prognostic Score (NPS) is a composite index of nutritional and inflammatory status that has demonstrated prognostic value across various critical illnesses. This study aimed to evaluate the prognostic significance of NPS in patients with acute pancreatitis (AP). **Methods:** In this retrospective cohort study, 452 patients diagnosed with AP between January 2024 and March 2026 were classified as mild (MAP), moderately severe (MSAP), or severe (SAP) according to the revised Atlanta classification. NPS was calculated from admission serum albumin, total cholesterol, neutrophil-to-lymphocyte ratio, and lymphocyte-to-monocyte ratio. **Results:** Of the 452 patients, 276 (61.1%) had MAP, 124 (27.4%) had MSAP, and 52 (11.5%) had SAP. NPS was significantly higher in SAP patients than in MAP and MSAP patients (for both comparisons; *p* < 0.001). ROC analysis showed an AUC of 0.758 (95% CI: 0.693–0.824) for predicting SAP, with an optimal cut-off of NPS ≥ 3 yielding a sensitivity of 80.8% and a specificity of 61.3%. Patients were stratified into low (0–2) and high (3–4) NPS groups. High NPS was significantly associated with SAP (χ^2^ = 33.044, *p* < 0.001), with a negative predictive value of 96.1%. In multivariable analysis, high NPS was an independent predictor of SAP (OR: 4.346, 95% CI: 2.018–9.362; *p* < 0.001). **Conclusions:** NPS, calculated from four routine laboratory parameters available at admission, is a simple, rapid, and independent predictor of disease severity in AP and may support early risk stratification in the emergency setting.

## 1. Introduction

Acute pancreatitis (AP) is a common gastrointestinal emergency with a clinical spectrum ranging from mild, self-limiting disease to life-threatening severe acute pancreatitis (SAP), which is associated with systemic organ failure and significant mortality [1,2]. Prompt and accurate stratification of disease severity at admission is essential for guiding therapeutic decisions and improving patient outcomes [3]. Although several multifactorial scoring systems, such as the Acute Physiology and Chronic Health Evaluation II (APACHE II), Bedside Index for Severity in Acute Pancreatitis (BISAP), and the Ranson score (RS), have been validated for this purpose, their complexity and the need for multiple time points limit their practical utility at the bedside [4,5]. Consequently, there is a need for a simpler, faster, real-time tool to predict disease prognosis at the time of hospital admission.

The Naples Prognostic Score (NPS), introduced by Galizia et al. [6], is a multidimensional composite score that includes total cholesterol, neutrophil-to-lymphocyte ratio (NLR), lymphocyte-to-monocyte ratio (LMR), and serum albumin concentrations to reflect systemic nutritional and inflammatory status. In recent years, its prognostic significance in pancreatic cancer [7], lung cancer [8], sepsis [9], heart failure [10], acute coronary syndrome [11], and chronic renal failure [12] has been investigated. Although recent studies have begun to examine NPS within the context of AP [13,14], its prognostic utility needs to be systematically characterized using a well-defined threshold, and its comparison with established severity scores is necessary.

In this study, we aimed to determine the prognostic significance of the NPS in patients with AP.

## 2. Materials and Methods

### 2.1. Patients and Study Design

**Figure 1:**
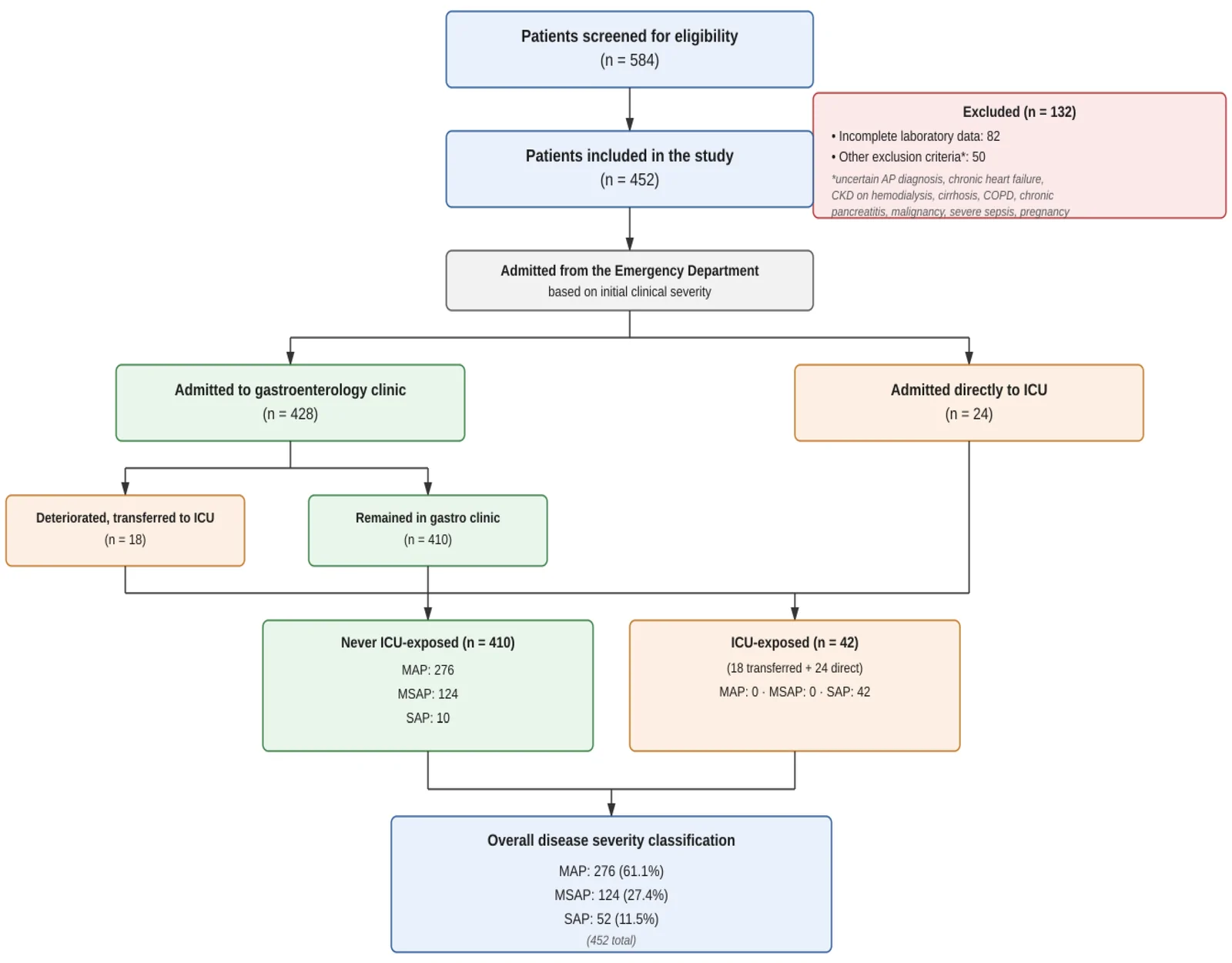
Patient flow diagram according to initial site of admission and final Atlanta severity classification. (ICU: Intensive Care Unit; MAP: mild acute pancreatitis; MSAP: moderately severe acute pancreatitis; SAP: severe acute pancreatitis).

The present study was a retrospective cohort study conducted at Antalya City Hospital. Patients aged 18 years and older who presented to the emergency department and were diagnosed with AP between January 2024 and March 2026 were screened for eligibility (*n* = 584). Patients were initially identified through ICD-coded diagnoses of acute pancreatitis in the hospital information system, and diagnoses were subsequently confirmed against the diagnostic criteria; patients who did not meet these criteria were excluded. Of these, 82 patients were excluded due to incomplete data (total cholesterol, neutrophil count, lymphocyte count, monocyte count, and albumin). An additional 50 patients were excluded for other reasons: uncertain diagnosis of AP (17 patients), chronic heart failure (6 patients), chronic renal failure requiring long-term hemodialysis (5 patients), cirrhosis (1 patient), chronic obstructive pulmonary disease (8 patients), chronic pancreatitis (6 patients), malignancy (4 patients), concomitant severe sepsis (2 patients), and pregnancy (1 patient). Finally, 452 patients diagnosed with AP were included in the study. For patients with repeated hospitalizations, only the first admission was included in the study. Patients included in the study were those who presented to the emergency department with a diagnosis of AP; patients were subsequently admitted to either the gastroenterology clinic or the ICU depending on their clinical status at the time of triage. We have illustrated this patient flow in a diagram (Figure 1).

Five scoring systems were used: RS, APACHE II, BISAP, SIRS and NPS. All scores were calculated based on laboratory test results and vital signs at admission to the emergency department.

### 2.2. Definitions

The diagnosis of AP was established based on a minimum of two of the following three criteria [15]: (1) typical girdle-type abdominal pain; (2) serum amylase and/or lipase levels exceeding three times the upper limit of normal; and (3) imaging results indicative of AP on contrast-enhanced computed tomography or ultrasound.

The revised Atlanta classification [15] defines mild acute pancreatitis (MAP) as the lack of organ failure and local or systemic complications. Moderately severe acute pancreatitis (MSAP) is defined by the occurrence of local or systemic complications or temporary organ failure lasting less than 48 h. Persistent organ failure lasting more than 48 h is defined as SAP.

Organ failure was assessed using the modified Marshall scoring system, in which each of the three organ systems—respiratory, renal, and cardiovascular—is scored from 0 to 4 based on the degree of dysfunction; a score of ≥2 in any single system was defined as organ failure [16]. The respiratory component was scored according to the PaO_2_ (mmHg)/FiO_2_ ratio: 0 = no arterial blood gas performed, 1 = 301–400, 2 = 201–300, 3 = 101–200, 4 = ≤100. The renal component was scored according to serum creatinine: 0 = ≤1.4 mg/dL, 1 = 1.4–1.8 mg/dL, 2 = 1.9–3.5 mg/dL, 3 = 3.6–4.9 mg/dL, 4 = ≥4.9 mg/dL. The cardiovascular component was scored according to systolic blood pressure (SBP) and fluid/inotrope responsiveness: 0 = SBP > 90 mmHg, 1 = SBP < 90 mmHg responsive to fluid resuscitation, 2 = SBP < 90 mmHg not fluid-responsive, 3 = SBP < 90 mmHg with pH < 7.3, 4 = SBP < 90 mmHg with pH < 7.2. Organ failure was reassessed at 48 h: resolution within 48 h was classified as transient organ failure (consistent with MSAP), whereas persistence beyond 48 h was classified as persistent organ failure (consistent with SAP), in accordance with the revised Atlanta classification.

Systemic Inflammatory Response Syndrome (SIRS) is characterized by the presence of two or more of the following criteria: Temperature exceeding 38 °C or below 36 °C, heart rate above 90 beats per minute, respiration rate exceeding 20 breaths per minute or partial pressure of CO_2_ below 32 mmHg, and leukocyte count exceeding 12,000 or below 4000/microliters or exceeding 10% immature forms [17].

Local complications included peripancreatic fluid collections, pseudocyst formation, pancreatic and peripancreatic necrosis, and walled-off necrosis. Death was defined as occurring during hospitalization [15].

NPS was calculated using four parameters defined in the original study: serum albumin (g/dL), total cholesterol (mg/dL), NLR, and LMR. Patients with NLR ≤ 2.96, LMR > 4.44, serum albumin ≥ 4.0 g/dL, and total cholesterol > 180 mg/dL were assigned 0 points, whereas those with NLR > 2.96, LMR ≤ 4.44, serum albumin < 4.0 g/dL, and total cholesterol ≤ 180 mg/dL were assigned 1 point for each parameter. The total NPS ranged from 0 to 4 [6]. NPS classification was divided into two groups: low (0–2) and high (3–4), as in the literature [18].

### 2.3. Statistics

Descriptive and comparative statistical analyses were performed using IBM SPSS Statistics for Windows, version 27.0 (IBM Corp., Armonk, NY, USA). Receiver operating characteristic (ROC) curve analyses, DeLong tests for pairwise AUC comparisons, and bootstrap-based internal validation were performed in R (version 4.6.1; R Foundation for Statistical Computing, Vienna, Austria) using the pROC package (version 1.19.0.1). Firth’s penalized-likelihood logistic regression was performed using the logistf package (version 1.26.1) in R. Variance inflation factors were calculated using the car package (version 3.1-5) in R. Categorical variables were expressed as frequencies and percentages [*n* (%)]. In contrast, continuous variables were reported as medians with interquartile ranges (IQRs). Normality of continuous variables was assessed using the Kolmogorov–Smirnov and Shapiro–Wilk tests, supplemented by visual inspection of histograms and Q-Q plots, as well as evaluation of skewness and kurtosis coefficients. Since none of the continuous variables were normally distributed, the Kruskal–Wallis test was used to compare the three severity groups, and the Chi-square test was used to compare categorical variables. Missing data were handled using a complete-case approach for each analysis. Patients with missing values for any of the four components required to calculate NPS (serum albumin, total cholesterol, neutrophil count, and lymphocyte/monocyte count) were excluded from the study at the outset, as detailed above. For secondary analyses involving variables that were not uniformly available at admission—including arterial blood gas parameters (PaO_2_, pH), lactate, and components of the APACHE II score, which are typically obtained only in patients with more severe clinical presentations or ICU admission—each analysis was restricted to patients with complete data for the variables involved, and the corresponding sample size is reported alongside each result. No imputation was performed for missing values. The diagnostic performance of NPS in predicting SAP was evaluated by ROC curve analysis, and the AUC was calculated with 95% confidence intervals (CIs). The optimal cut-off value was determined by maximizing the Youden index. Internal validation of the NPS cut-off was performed using bootstrap resampling (B = 10,000 iterations), in which the optimal Youden cut-off was re-derived in each resample and the corresponding optimism in AUC was estimated following Harrell’s bootstrap optimism-correction approach. To compare the discriminative performance of NPS with established prognostic scores (SIRS, BISAP, Ranson, and APACHE II), pairwise comparisons of AUCs were performed using DeLong’s test, restricted to patients with complete data for all five scores; *p*-values were adjusted for multiple comparisons using the Holm method. Candidate predictors for univariable analysis were selected a priori based on their established clinical relevance to AP severity, as previously reported in the literature. These included age, sex, serum creatinine, glucose, CRP, SIRS score, BISAP score, Ranson score, APACHE II score, and NPS. Conventional maximum-likelihood logistic regression produced unstable estimates for SIRS, BISAP, Ranson, and APACHE II scores, attributable to quasi-complete separation resulting from the low number of SAP events in certain subgroups; univariable associations for these four scores were therefore estimated using Firth’s penalized-likelihood logistic regression, which yields stable and unbiased estimates under these conditions. Univariable associations for the remaining candidate predictors (age, sex, creatinine, glucose, CRP, and NPS), for which conventional logistic regression converged without evidence of instability, are reported using standard maximum-likelihood estimates, consistent with the multivariable model. Variables with *p* < 0.05 in univariable analysis were subsequently entered into the multivariable model, together with age, which was retained a priori as a clinically relevant covariate regardless of its univariable significance. The multivariable model was fitted using conventional maximum-likelihood logistic regression, which converged without evidence of separation. Multicollinearity among covariates was assessed using variance inflation factors (VIFs), with VIFs > 5 considered indicative of significant multicollinearity. Results were expressed as odds ratios (ORs) with 95% CIs. A two-tailed *p*-value of less than 0.05 was considered statistically significant for all analyses.

## 3. Results

The study comprised 452 patients diagnosed with AP. The demographic and baseline laboratory characteristics of the patients are presented in Table 1. The mean age of the cohort was 58 ± 18 years. The gender distribution was nearly equal, with 233 females (51.5%) and 219 males (48.5%). The most common etiology was biliary pancreatitis, accounting for 328 patients (72.6%), followed by alcohol (8.6%). Among the 452 patients, 276 (61.1%) were classified as MAP, 124 (27.4%) as MSAP, and 52 (11.5%) as SAP according to the revised Atlanta classification. Table 2A demonstrates the basic demographic, clinical, and etiological characteristics according to the Revised Atlanta Classification.

White blood cell count and neutrophil count at admission were significantly higher with increasing disease severity (*p* < 0.001 for both). Platelet count differed significantly across groups (*p* = 0.045), whereas lymphocyte count and monocyte count did not (*p* = 0.124 and *p* = 0.557, respectively). CRP levels at admission, 24 h and 72 h were significantly higher in SAP patients (*p* < 0.001 for all comparisons). BUN and creatinine at admission were significantly elevated in SAP patients (*p* = 0.001 and *p* < 0.001, respectively), and this pattern persisted at 24 and 72 h (*p* < 0.001 for all). Serum albumin at admission was significantly lower in the SAP group (*p* = 0.005), with a further decline observed at 24 and 72 h (*p* < 0.001 for both). Total cholesterol levels also differed significantly across groups (*p* = 0.009) (Table 2B).

Length of hospital stay increased significantly with disease severity, with SAP patients having the longest median stay of 7 days (IQR 4–10) compared to 6 days (IQR 4–9) in MSAP and 4 days (IQR 3–6) in MAP (*p* < 0.001). ICU admission was required in 80.8% of SAP patients, while no MAP or MSAP patients required ICU care (*p* < 0.001). In-hospital mortality occurred exclusively in the SAP group, affecting 9 patients (17.3%) (*p* < 0.001). BISAP and APACHE II scores were significantly higher in the SAP group (*p* < 0.001 for both), whereas the Ranson score did not differ significantly across groups (*p* = 0.349). SIRS score also differed significantly across severity groups (*p* = 0.011) (Table 2C).

NPS was significantly higher in SAP patients than in the MAP and MSAP groups (median 3.00 [IQR 3.00–4.00] vs. 2.00 [IQR 2.00–3.00] and 2.00 [IQR 2.00–3.00], respectively; *p* < 0.001). In the MAP group, the majority of patients had an NPS of 2 (42.8%), while scores of 0 and 1 were present in 9.8% and 12.0%, respectively. In the SAP group, higher NPS values predominated: 44.2% of patients had a score of 3 and 36.5% had a score of 4 (Table 2B).

**Figure 2:**
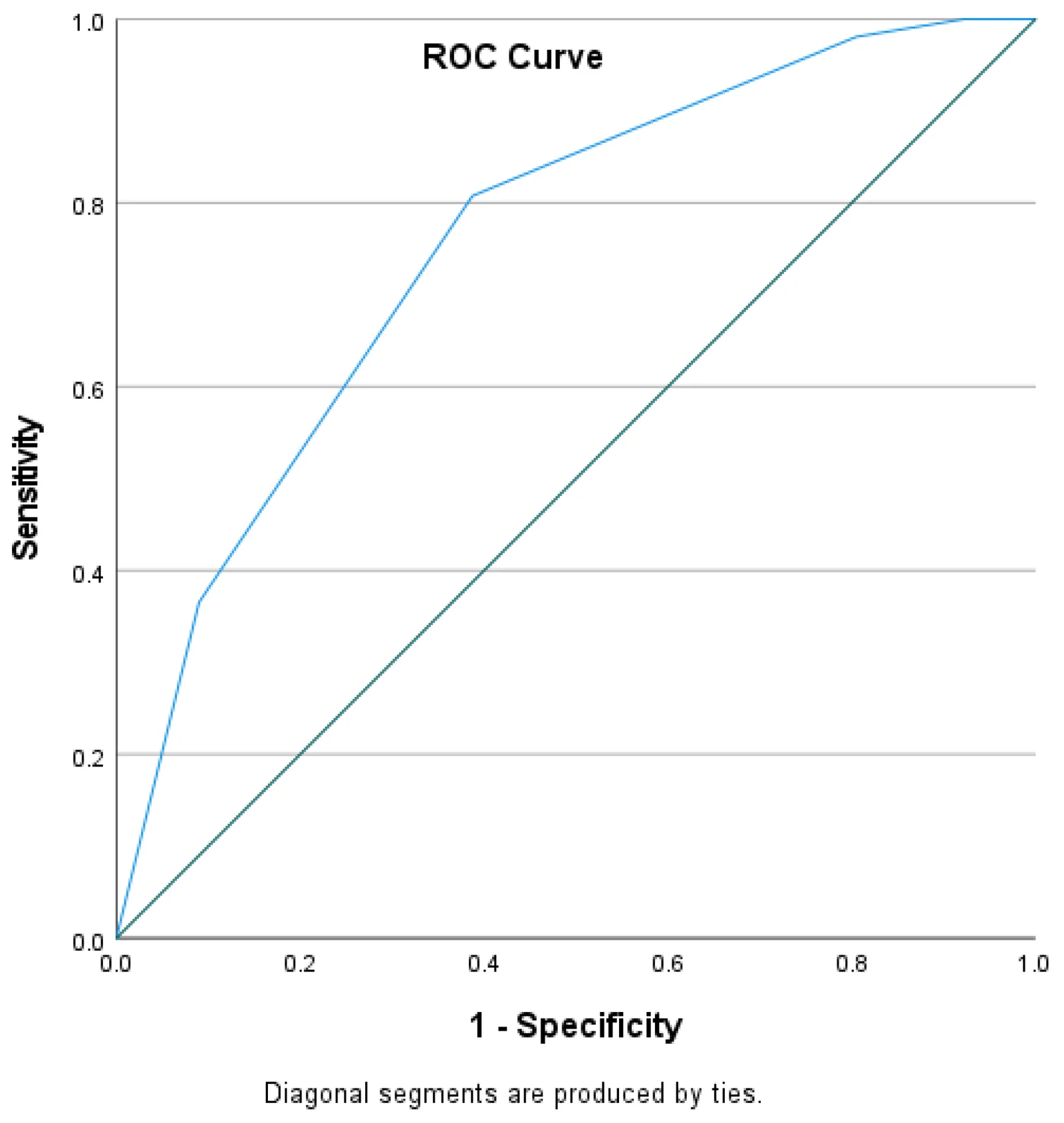
Receiver operating characteristic curve for the NPS to predict severe acute pancreatitis. The blue line represents the ROC curve for NPS; the green diagonal line represents the reference line of no discrimination (AUC = 0.5).

ROC curve analysis was conducted to assess the diagnostic performance of NPS in predicting SAP. The AUC for NPS was 0.758 (95% CI: 0.693–0.824; *p* < 0.001), indicating acceptable discrimination for identifying SAP among patients with AP. The optimal cut-off value was an NPS ≥ 3, determined by maximizing the Youden index, yielding a sensitivity of 80.8% and a specificity of 61.3% (Figure 2). Internal validation using bootstrap resampling (B = 10,000) yielded an optimism-corrected AUC of 0.758, which was essentially identical to the apparent AUC (mean optimism = 0.0001), indicating minimal overfitting. The optimal cut-off (NPS ≥ 3) was selected in 97.7% of bootstrap resamples, confirming the stability of this threshold.

When patients were stratified into low NPS (0–2) and high NPS (3–4), 197 patients (43.6%) were classified as having high NPS. Among SAP patients, 42 of 52 (80.8%) had a high NPS, whereas among MAP and MSAP patients combined, 155 of 400 (38.8%) had a high NPS. The association between the NPS and disease severity was statistically significant (χ^2^ = 33.044, *p* < 0.001). The positive predictive value was 21.3%, and the negative predictive value was 96.1%, suggesting that a low NPS effectively rules out severe disease.

Univariable logistic regression identified creatinine (OR: 2.802, 95% CI: 1.548–5.074; *p* < 0.001), CRP (OR: 1.006, 95% CI: 1.003–1.009; *p* < 0.001), glucose (OR: 1.004, 95% CI: 1.000–1.008; *p* = 0.032), and high NPS (OR: 6.639, 95% CI: 3.237–13.617; *p* < 0.001) as significant predictors of SAP, whereas age did not reach statistical significance (*p* = 0.274). In the multivariable logistic regression model incorporating all variables significant in univariable analysis, creatinine (OR: 2.038, 95% CI: 1.264–3.287; *p* = 0.003), CRP (OR: 1.004, 95% CI: 1.001–1.007; *p* = 0.004), glucose (OR: 1.004, 95% CI: 1.000–1.008; *p* = 0.048), and high NPS (OR: 4.346, 95% CI: 2.018–9.362; *p* < 0.001) were all independently associated with SAP. These results are presented in Table 3.

Multicollinearity among covariates in the multivariable model was assessed using variance inflation factors (VIFs); all VIFs ranged from 1.008 to 1.164 (age: 1.047; creatinine: 1.061; CRP: 1.104; glucose: 1.008; NPS: 1.164), indicating no evidence of significant multicollinearity (Supplementary Table S1). A likelihood ratio test comparing the full multivariable model with a reduced model excluding NPS confirmed that NPS contributed significant independent information beyond age, creatinine, CRP, and glucose (χ^2^ = 15.81, df = 1, *p* < 0.001).

To formally compare the discriminative performance of NPS with established prognostic scores, we performed a sensitivity analysis restricted to patients with complete data for all five scores (SIRS, BISAP, Ranson, APACHE II, and NPS; *n* = 60, of whom 20 [33.3%] had SAP). AUCs were 0.835 (95% CI: 0.723–0.928) for NPS, 0.817 (95% CI: 0.696–0.918) for APACHE II, 0.692 (95% CI: 0.556–0.816) for BISAP, 0.662 (95% CI: 0.521–0.787) for SIRS, and 0.657 (95% CI: 0.511–0.795) for Ranson. On pairwise DeLong testing, NPS showed a numerically higher AUC than all other scores; unadjusted *p*-values suggested a nominally significant difference versus SIRS (*p* = 0.031) and BISAP (*p* = 0.048), but no difference remained statistically significant after adjustment for multiple comparisons (all adjusted *p* > 0.05), including the comparison with APACHE II (*p* = 0.762) (Supplementary Table S2).

## 4. Discussion

In the present study, we investigated the prognostic value of the NPS in patients with AP and demonstrated that NPS was significantly associated with disease severity. Two recent studies have examined NPS in the context of AP. Zhang et al. [13] evaluated NLR, LMR, and NPS among 417 patients with AP and incorporated these markers into a machine learning model for predicting severity and short-term prognosis. Çelikdelen et al. [14] assessed NPS alongside several inflammatory, nutritional, and composite indices in 348 patients with AP; although NPS was associated with outcomes in unadjusted analyses, it did not retain independent prognostic significance in their multivariable models. In contrast to these studies, the present analysis specifically defined and internally validated an NPS threshold (≥3) for predicting SAP, using bootstrap resampling to assess its stability, and directly compared its discriminative performance with established severity scores, including SIRS, BISAP, Ranson, and APACHE II.

In this study, biliary etiology was the most common cause of AP (72.6%), followed by alcohol (8.6%). This etiological distribution is consistent with epidemiological data in the literature, in which gallstone-related pancreatitis accounts for approximately 40–70% of all AP cases. Our cohort’s overall SAP rate was 11.5%, which falls within the reported range of 10–20% [1,2]. In-hospital mortality was 2.0% (9/452), occurring exclusively in SAP patients (17.3%), which is consistent with the well-established association between organ failure and mortality in AP [1,3].

The NPS is a composite score incorporating four readily available laboratory parameters: serum albumin, total cholesterol, NLR, and LMR. Each of these parameters reflects the pathophysiological pathways related to the systemic inflammatory response observed in AP. Serum albumin is a well-established marker of nutritional status and systemic inflammation. Hypoalbuminemia in AP arises from increased vascular permeability, third-space fluid shifts, and impaired hepatic synthesis in the context of systemic inflammatory response syndrome and has been previously associated with disease severity and mortality in AP [3]. Similarly, hypocholesterolemia has been recognized as a marker of poor prognosis in critical illness, as cholesterol is essential for cell membrane integrity and immune cell function; reduced levels reflect impaired hepatic synthetic capacity and systemic metabolic dysregulation [19,20]. The relationship between serum cholesterol and AP severity has been examined previously: Hong et al. [21] reported a U-shaped association between admission total cholesterol and severe AP in 648 patients, with both low and high concentrations associated with increased risk, while Wang et al. [22] identified total cholesterol as a predictor of in-hospital mortality among 338 patients with severe AP. The present study extends this literature by incorporating cholesterol as one component of a composite score (NPS) alongside albumin, NLR, and LMR, rather than evaluating it in isolation, thereby capturing its combined contribution to prognosis in AP together with nutritional and inflammatory status. NLR, reflecting the balance between innate inflammatory drive and adaptive immune response, has emerged as a robust prognostic marker across a wide range of inflammatory conditions [23]. Elevated NLR in AP indicates neutrophil-dominant systemic inflammation with relative lymphopenia, a pattern associated with more severe disease courses and mortality [24,25]. LMR, on the other hand, captures the relationship between lymphocyte-mediated adaptive immunity and monocyte-driven innate inflammation; a low LMR suggests immune dysregulation and has been associated with worse outcomes in AP [26,27]. By integrating these four parameters into a single composite score, NPS offers a multidimensional perspective on the nutritional and inflammatory context at hospital admission, making it a practical and biologically significant tool for early risk stratification in AP.

The prognostic utility of NPS has been investigated across a broad spectrum of clinical conditions. It was originally introduced by Galizia et al. [6] in 2017 as an independent predictor of long-term outcome in patients undergoing surgery for colorectal cancer, where higher NPS was associated with significantly worse survival. Subsequently, its prognostic significance has been demonstrated in various oncological and non-oncological settings. In the oncological field, Wu et al. [7] recently reported that an NPS-based nomogram enhanced perioperative risk stratification in resected pancreatic cancer, highlighting its value in predicting postoperative complications and survival. Similarly, a meta-analysis by Liu et al. [8] confirmed the prognostic role of NPS in lung cancer, with higher scores correlating with poorer overall survival. Moreover, NPS has shown prognostic value in critically ill patients. Chen et al. [9] demonstrated that an inflammatory-nutritional index model incorporating NPS components provided early prognostic prediction in sepsis. In the cardiovascular domain, Wang et al. [10] reported a significant association between high NPS and increased all-cause and cardiovascular mortality in patients with heart failure, while Özen et al. [11] found that elevated NPS was associated with worse clinical outcomes following percutaneous coronary intervention for acute coronary syndrome. Most recently, Avcı et al. [12] demonstrated the predictive value of NPS for rapid kidney function decline in stage 3–4 chronic kidney disease. Collectively, these findings demonstrated that NPS captures a clinically significant signal in various disease states characterized by systemic inflammation and nutritional deficiencies; this pathophysiological profile also plays a central role in the progression of AP. Our study further strengthened this evidence by demonstrating that NPS is a valid prognostic tool in AP.

In our study, NPS achieved an AUC of 0.758 for predicting SAP, with an optimal cut-off of ≥3. These results should be interpreted with appropriate caution. An AUC of 0.758, together with a sensitivity of 80.8% and a specificity of 61.3%, reflects only fair-to-modest discriminatory performance by conventional standards and is notably lower than the discriminatory performance originally reported for NPS in its validation cohort of patients undergoing surgery for colorectal cancer. This difference likely reflects fundamental distinctions between the two clinical contexts. NPS was originally developed to predict long-term oncological survival in a relatively stable, preoperative surgical population, in which nutritional and immune-inflammatory status largely reflect chronic, cumulative processes such as tumor burden and cachexia. In contrast, AP is an acute inflammatory condition in which the four NPS components—particularly albumin and the leukocyte-based ratios—can fluctuate rapidly within hours due to capillary leak, third-space fluid shifts, and acute-phase reactant kinetics. It may therefore be less stable and less reflective of overall disease trajectory than in the oncological setting for which NPS was originally designed. Furthermore, AP severity is determined not only by nutritional-inflammatory status but also by disease-specific factors such as local complications (necrosis, fluid collections) and organ-specific dysfunction, which NPS does not directly capture. These differences may explain, at least in part, why the discriminatory performance of NPS in our AP cohort was more modest than in its original oncological validation, and underscore that NPS should be regarded as a complementary rather than a standalone prognostic tool in this setting. The high negative predictive value of 96.1% suggests that a low NPS at admission effectively identifies patients unlikely to develop severe disease. However, predictive values are inherently dependent on disease prevalence, and the low prevalence of SAP in our cohort (11.5%) likely contributed to this high NPV; in populations with a higher SAP prevalence, the NPV of NPS would be expected to be lower. Furthermore, as this retrospective study did not evaluate clinical management strategies or patient outcomes based on NPS-guided decision-making, any potential role for NPS in supporting decisions such as early discharge or step-down care remains hypothesis-generating and would require prospective validation before clinical implementation. While the positive predictive value was relatively modest (21.3%), this is expected given the inherently low prevalence of SAP in the overall AP population. Beyond its discriminatory performance in ROC analysis, NPS also emerged as an independent predictor of SAP in multivariable logistic regression analysis. After adjusting for creatinine, CRP, and glucose—all of which are established markers of AP severity—high NPS (≥3) retained a significant and strong independent association with SAP (OR: 4.346). This finding supports the notion that the prognostic value of NPS was independent of other factors in predicting AP severity. The magnitude of this relationship, with an odds ratio exceeding four, suggests potential clinical utility of NPS as a triage tool in the emergency department. However, prospective validation is needed before firm clinical recommendations can be made.

When formally compared with established prognostic scores using DeLong tests in the subgroup of patients with complete data for all scores (*n* = 60), NPS showed the numerically highest AUC among all scores evaluated, including APACHE II, BISAP, SIRS, and Ranson. Although unadjusted comparisons suggested a nominal advantage of NPS over SIRS and BISAP, these differences did not survive correction for multiple comparisons, and no score—including APACHE II—differed significantly from NPS. Given the small size of this subgroup (*n* = 60) and the correspondingly wide confidence intervals, these findings should be interpreted as hypothesis-generating rather than conclusive evidence of superiority. Nonetheless, in this small complete-case subgroup, no statistically significant difference in AUC was demonstrated between NPS and the established scoring systems evaluated; this should not be interpreted as evidence of equivalence or non-inferiority, given the limited statistical power to detect such differences. This subgroup—restricted to patients in whom APACHE II could be calculated—also had a substantially higher SAP prevalence (33.3%) than the overall cohort (11.5%), indicating a more severely ill population and limiting the generalizability of this comparison.

Despite its modest discriminatory performance, a key strength of NPS as a prognostic tool in AP lies in its practical simplicity. Unlike APACHE II, which requires 12 physiological and laboratory variables and is primarily designed for ICU-based assessment, or the Ranson score, which necessitates data collection at two separate time points, NPS can be calculated from a single routine blood sample obtained at the time of emergency department admission. The four parameters comprising NPS—serum albumin, total cholesterol, NLR, and LMR—are all derived from standard complete blood count and biochemistry panels that are universally available in emergency settings, without the need for additional tests or repeated measurements. This characteristic makes NPS particularly well-suited for real-time risk stratification at the point of first clinical contact, before the full clinical picture has emerged. In our study, all ICU admissions and deaths occurred within the SAP group, underscoring the clinical importance of early identification and prompt intervention in these patients. Early diagnosis of SAP patients, timely initiation of aggressive fluid resuscitation, and appropriate escalation of care are also crucial; all of these have been shown to improve outcomes in severe disease [1,3]. Conversely, accurate identification of low-risk patients—facilitated by the high negative predictive value of NPS observed in our study—may potentially support strategies such as reduced-intensity monitoring in resource-limited settings; however, this application was not directly evaluated in the present study and would require prospective validation before being incorporated into clinical decision-making.

The present study has limitations that should be acknowledged. The retrospective, single-center design and predominance of biliary etiology (72.6%) may limit the generalizability of our findings to institutions with different etiological distributions. Prospective multicenter studies are needed to validate the prognostic utility of NPS in AP across diverse clinical settings. The availability of certain parameters such as PaO_2_, lactate, arterial pH, and APACHE II score was limited to subsets of patients; a complete-case approach was used throughout, with no imputation for missing values. As a result, analyses involving these variables (e.g., APACHE II, available in only 64 of 452 patients) were based on smaller and potentially non-representative subsets of the cohort, which may have introduced selection bias. With 52 SAP events and five predictors in the final multivariable model, the events-per-variable ratio (approximately 10.4) was near the conventional minimum threshold recommended for stable logistic regression estimates; this should be considered when interpreting the precision of the multivariable odds ratios. While NPS was calculated using admission laboratory values as per the original definition, the optimal timing of NPS assessment in AP—whether at admission, 24 h, or 48 h—has not been systematically evaluated. Serial NPS measurements at multiple time points may provide additional prognostic information beyond the single admission value assessed in this study. Detailed clinical and laboratory data for the 82 excluded patients were not retrievable from the dataset, precluding a formal comparison between included and excluded patients. Although the NPS cut-off was derived and evaluated within the same cohort, raising a theoretical risk of overfitting, bootstrap-based internal validation demonstrated negligible optimism (corrected AUC = 0.758) and high stability of the selected threshold (selected in 97.7% of 10,000 resamples), supporting the robustness of this cut-off within our population. External validation in independent cohorts remains necessary to confirm generalizability. Finally, as cholesterol levels may be influenced by dietary habits, lipid-lowering medications, and comorbid metabolic conditions, these factors were not systematically controlled for in the present analysis and may have introduced confounding in the NPS calculation.

## 5. Conclusions

In conclusion, the present study demonstrates that the NPS is a significant and independent predictor of disease severity in AP. NPS was significantly higher in patients with SAP than in those with MAP and MSAP, and an NPS ≥ 3 at admission yielded an AUC of 0.758, with a sensitivity of 80.8% and a negative predictive value of 96.1% for predicting SAP. These findings suggest that NPS, calculated from four routine laboratory parameters available at the time of initial presentation, can serve as a simple, rapid, and independent tool for early risk stratification in AP.

## Figures and Tables

**Table 1:** Patient Demographic and Clinical Characteristics.

		*n*	%/Mean ± SD
Age		452	58 ± 18
**Gender**	Female	233	51.5%
	Male	219	48.5%
Pain		439	97.1%
Inflammation on Imaging		233	51.5%
**Etiology**	Biliary	328	72.6%
	Alcohol	39	8.6%
	Idiopathic	35	7.7%
	Post-ERCP	25	5.5%
	Hypertriglyceridemia	18	4.0%
	Autoimmune	3	0.7%
	Drug-induced	2	0.4%
	Hypercalcemia	2	0.4%
**Comorbidities**	Hypertension (HT)	179	39.6%
	Diabetes Mellitus (DM)	125	27.7%
	Coronary Artery Disease (CAD)	55	12.2%
	Chronic Kidney Disease (CKD)	17	3.8%

HT: Hypertension; DM: Diabetes Mellitus; CAD: Coronary Artery Disease; CKD: Chronic Kidney Disease; *n*: number of patients; SD: standard deviation. Continuous variables are presented as mean ± standard deviation (Mean ± SD), and categorical variables are presented as number (*n*) and percentage (%). Column headers are bold for visual distinction. Bold text indicates variable category headers.

**Table 2:** (**A**) Baseline demographic, clinical, and etiological characteristics according to the revised Atlanta classification. (**B**) Laboratory parameters and prognostic scoring systems according to the revised Atlanta classification. (**C**) Local complications, interventions, and clinical outcomes according to the revised Atlanta classification.

(**A**)				
**Category**	**MAP (*n* = 276)**	**MSAP (*n* = 124)**	**SAP (*n* = 52)**	***p***
**Categorical variables**				
**Gender**				
Female	137 (49.6%)	68 (54.8%)	28 (53.8%)	0.591
Male	139 (50.4%)	56 (45.2%)	24 (46.2%)	
Pain	268 (97.1%)	121 (97.6%)	50 (96.2%)	0.874
Imaging Performed	258 (93.5%)	123 (99.2%)	48 (92.3%)	**0.037**
Inflammation on Imaging	98 (35.5%)	99 (79.8%)	36 (69%)	**<0.001**
**Etiology**				
Biliary	204 (73.9%)	88 (71.0%)	36 (69.2%)	**0.002**
Alcohol	24 (8.7%)	7 (5.6%)	8 (15.4%)	
Idiopathic	19 (6.9%)	15 (12.1%)	1 (1.9%)	
Drug	2 (0.7%)	0 (0.0%)	0 (0.0%)	
Post-ERCP	20 (7.2%)	3 (2.4%)	2 (3.8%)	
Hypertriglyceridemia	5 (1.8%)	10 (8.1%)	3 (5.8%)	
Hypercalcemia	2 (0.7%)	0 (0.0%)	0 (0.0%)	
Autoimmune	0 (0.0%)	1 (0.8%)	2 (3.8%)	
Hypertension	109 (39.5%)	45 (36.3%)	25 (48.1%)	0.344
Diabetes Mellitus	70 (25.4%)	39 (31.5%)	16 (30.8%)	0.392
Coronary Artery Disease	36 (13.0%)	10 (8.1%)	9 (17.3%)	0.179
Chronic Kidney Disease	4 (1.4%)	4 (3.2%)	9 (17.3%)	**<0.001**
Chronic Liver Disease	4 (1.4%)	3 (2.4%)	1 (1.9%)	0.790
Chronic Respiratory Disease	14 (5.1%)	4 (3.2%)	1 (1.9%)	0.476
Malignancy	5 (1.8%)	7 (5.6%)	3 (5.8%)	0.081
**Continuous variables—Median (IQR)**				
Age (Years)	61 (46–72)	52 (38–66)	62 (49–71)	**0.020**
Systolic BP (mmHg)	135 (123–148)	133 (120–156)	142 (109–163)	0.846
Diastolic BP (mmHg)	78 (70–85)	76 (66–85)	75 (63–87)	0.281
Heart Rate	83 (74–92)	81 (74–90)	87 (74–97)	0.203
Respiratory Rate	19 (18–20)	18 (18–20)	18 (18–20)	0.260
Temperature	36.1 (36.0–36.5)	36.2 (36.0–36.5)	36.0 (36.0–36.5)	0.886
SpO_2_	98 (96–98)	98 (98–99)	98 (97–99)	**0.011**
(**B**)				
**Category**	**MAP (*n* = 276)**	**MSAP (*n* = 124)**	**SAP (*n* = 52)**	***p***
**Laboratory parameters at admission—Median (IQR)**				
PaO_2_ (mmHg)	91.0 (87.0–95.0)	89.0 (78.0–93.0)	81.0 (73.5–93.0)	**<0.001**
Lactate (mmol/L)	1.6 (1.0–2.2)	1.8 (1.2–2.1)	2.0 (1.4–4.0)	**0.025**
WBC (×10^9^/µL)	11.01 (8.82–13.61)	12.90 (10.80–15.30)	14.20 (10.55–16.69)	**<0.001**
Neutrophil (×10^9^/µL)	8.72 (6.02–11.32)	10.55 (8.22–12.89)	12.00 (8.85–14.31)	**<0.001**
LYM (×10^9^/µL)	1.50 (0.91–1.94)	1.72 (1.23–2.00)	1.45 (0.89–2.09)	0.124
Monocyte (×10^9^/µL)	0.66 (0.47–0.89)	0.70 (0.48–0.92)	0.68 (0.50–0.89)	0.557
PLT (×10^9^/µL)	251.5 (196.5–296.0)	274.5 (202.5–312.5)	240.5 (180.0–279.0)	**0.045**
Hb (g/dL)	13.6 (12.3–14.8)	13.6 (12.5–15.1)	12.6 (11.2–15.1)	0.404
Hematocrit (%)	41.0 (37.6–44.3)	41.1 (38.5–43.7)	39.2 (35.6–45.2)	0.748
CRP (mg/L)	22.20 (8.40–76.50)	23.13 (10.25–78.50)	88.70 (25.65–193.15)	**<0.001**
BUN (mg/dL)	15.15 (11.85–21.00)	15.60 (10.60–26.80)	25.00 (14.60–40.00)	**0.001**
Creatinine (mg/dL)	0.86 (0.71–1.01)	0.86 (0.74–1.07)	1.07 (0.84–1.67)	**<0.001**
Albumin (g/dL)	4.0 (3.7–4.4)	4.0 (3.5–4.3)	3.8 (3.5–4.1)	**0.005**
Sodium (mEq/L)	139 (137–141)	137 (135–140)	137 (135–139)	**<0.001**
Potassium (mEq/L)	4.1 (3.8–4.4)	4.1 (3.8–4.5)	4.2 (3.9–4.7)	0.253
Chloride (mEq/L)	104.0 (101.0–106.0)	102.0 (99.0–105.0)	102.0 (99.0–104.0)	**0.003**
Calcium (mg/dL)	9.2 (8.9–9.7)	9.1 (8.8–9.7)	9.1 (8.2–9.6)	0.063
Glucose (mg/dL)	122 (101–146)	131 (107–184)	152 (111–207)	**0.001**
AST (U/L)	136 (38–305)	84 (28–255)	92 (29–256)	0.307
ALT (U/L)	162 (40–386)	88 (28–284)	59 (21–232)	**0.003**
ALP (U/L)	133.0 (83.0–215.0)	104.0 (66.0–179.0)	101.5 (65.0–156.0)	**<0.001**
GGT (U/L)	271 (87–551)	162 (48–411)	208 (56–297)	**0.005**
Total Bilirubin (mg/dL)	1.64 (0.88–3.19)	1.11 (0.78–2.25)	1.11 (0.80–2.48)	**0.006**
Direct Bilirubin (mg/dL)	0.700 (0.280–2.040)	0.370 (0.180–1.350)	0.430 (0.190–1.500)	**0.001**
Amylase (U/L)	599 (174–1520)	764 (233–1973)	579 (201–1454)	0.334
Lipase (U/L)	909.0 (222.0–2529.0)	1454.4 (476.4–2757.0)	1252.0 (320.0–1916.0)	0.115
Triglyceride (mg/dL)	97 (65–149)	100 (70–196)	143 (93–753)	**0.003**
LDL (mg/dL)	100 (78–126)	103 (67–129)	69 (45–112)	**0.010**
Total Cholesterol (mg/dL)	168 (139–203)	186 (150–227)	178 (122–219)	**0.009**
pH	7.400 (7.380–7.430)	7.390 (7.340–7.440)	7.345 (7.300–7.400)	**0.001**
LDH (U/L)	282 (218–413)	293 (206–520)	313 (208–519)	0.237
**Serial laboratory parameters at 24 h and 72 h—Median (IQR)**				
WBC at 24 h (×10^9^/µL)	8.72 (6.52–12.30)	11.50 (8.20–15.50)	12.60 (9.50–16.30)	**<0.001**
WBC at 72 h (×10^9^/µL)	8.00 (6.20–11.70)	8.70 (6.64–13.40)	10.31 (7.00–14.66)	**0.043**
Neutrophil at 24 h (×10^9^/µL)	6.36 (3.98–9.90)	8.22 (5.40–11.73)	10.15 (6.60–13.22)	**<0.001**
Neutrophil at 72 h (×10^9^/µL)	5.46 (3.52–9.00)	6.36 (4.30–10.20)	6.99 (4.90–12.50)	**0.007**
Lymphocyte at 24 h (×10^9^/µL)	1.570 (1.010–2.060)	1.480 (1.040–2.000)	1.500 (0.940–2.000)	0.604
Lymphocyte at 72 h (×10^9^/µL)	1.587 (1.190–2.030)	1.600 (1.200–2.000)	1.435 (0.820–1.900)	0.246
Platelet at 24 h (×10^9^/µL)	215 (172–266)	243 (173–291)	201 (169–299)	0.284
Platelets at 72 h (×10^9^/µL)	214 (167–269)	236 (189–294)	202 (163–295)	**0.039**
CRP at 24 h (mg/L)	69.70 (18.60–152.40)	129.00 (50.50–203.40)	169.10 (97.30–269.20)	**<0.001**
CRP at 72 h (mg/L)	66.70 (20.45–153.50)	108.35 (49.55–192.10)	153.85 (68.40–227.00)	**<0.001**
BUN at 24 h (mg/dL)	13.20 (9.20–19.70)	14.00 (8.60–22.30)	21.25 (14.15–33.10)	**<0.001**
BUN at 72 h (mg/dL)	11.00 (7.90–17.70)	10.20 (6.80–17.90)	17.90 (10.60–25.80)	**0.001**
Creatinine at 24 h (mg/dL)	0.80 (0.67–0.94)	0.83 (0.66–0.94)	0.96 (0.80–1.70)	**<0.001**
Creatinine at 72 h (mg/dL)	0.77 (0.68–0.90)	0.76 (0.67–0.90)	0.90 (0.71–1.91)	**0.002**
Albumin at 24 h (g/dL)	3.6 (3.3–3.8)	3.4 (3.1–3.7)	3.4 (3.0–3.7)	**<0.001**
Albumin at 72 h (g/dL)	3.5 (3.2–3.8)	3.2 (2.9–3.5)	3.1 (2.8–3.6)	**<0.001**
Hematocrit at 48 h (%)	37.4 (34.2–40.5)	35.3 (32.4–39.3)	33.7 (31.0–38.7)	**0.002**
**Prognostic scoring systems—Median (IQR)**				
SIRS Score	1 (0–1)	1 (0–1)	1 (0–2)	**0.011**
BISAP Score	1 (0–1)	1 (0–1)	1 (1–2)	**<0.001**
Ranson Score	1 (1–2)	2 (1–2)	2 (1–3)	0.349
APACHE II Score	11 (8–13)	10 (8–12)	17 (12–22)	**<0.001**
NPS	2.00 (2.00–3.00)	2.00 (2.00–3.00)	3.00 (3.00–4.00)	**<0.001**
**NPS distribution—*n* (%)**				
0	27 (9.8%)	4 (3.2%)	0 (0.0%)	**<0.001**
1	33 (12.0%)	14 (11.3%)	1 (1.9%)	
2	118 (42.8%)	49 (39.5%)	9 (17.3%)	
3	83 (30.1%)	36 (29.0%)	23 (44.2%)	
4	15 (5.4%)	21 (16.9%)	19 (36.5%)	
(**C**)				
**Category**	**MAP (*n* = 276)**	**MSAP (*n* = 124)**	**SAP (*n* = 52)**	***p***
**Local complications and interventions—*n* (%)**				
Acute Fluid Collection	0 (0.0%)	102 (82.3%)	12 (23.1%)	**<0.001**
Pancreatic Necrosis	0 (0.0%)	30 (24.2%)	9 (17.3%)	**<0.001**
Pseudocyst	0 (0.0%)	10 (8.1%)	5 (9.6%)	**<0.001**
Pancreatic WON	0 (0.0%)	8 (6.5%)	2 (3.8%)	**<0.001**
GI Bleeding	0 (0.0%)	1 (0.8%)	1 (1.9%)	0.123
ERCP Performed	126 (45.7%)	35 (28.2%)	14 (26.9%)	**0.001**
ICU Admission	0 (0.0%)	0 (0.0%)	42 (80.8%)	**<0.001**
In-hospital Mortality	0 (0.0%)	0 (0.0%)	9 (17.3%)	**<0.001**
30-day Readmission	3 (1.1%)	3 (2.4%)	1 (1.9%)	0.591
Altered Mental Status	6 (2.2%)	7 (5.6%)	16 (30.8%)	**<0.001**
Pleural Effusion	11 (4.0%)	7 (5.6%)	6 (11.5%)	0.082
**Clinical outcomes—Median (IQR)**				
Length of Stay (Days)	4 (3–6)	6 (4–9)	7 (4–10)	**<0.001**
Glasgow Coma Scale	15 (15–15)	15 (15–15)	15 (13–15)	**<0.001**

MAP: Mild Acute Pancreatitis; MSAP: Moderately Severe Acute Pancreatitis; SAP: Severe Acute Pancreatitis; ERCP: Endoscopic Retrograde Cholangiopancreatography; BP: Blood Pressure; SpO_2_: Peripheral Oxygen Saturation; IQR: Interquartile Range. Categorical variables are presented as number (%); continuous variables are presented as median (IQR). *p*-values were calculated using the appropriate statistical test for comparisons among the three groups. PaO_2_: Partial Pressure of Oxygen; WBC: White Blood Cell Count; LYM: Lymphocyte Count; PLT: Platelet Count; CRP: C-Reactive Protein; BUN: Blood Urea Nitrogen; AST: Aspartate Aminotransferase; ALT: Alanine Aminotransferase; ALP: Alkaline Phosphatase; GGT: Gamma-Glutamyl Transferase; LDL: Low-Density Lipoprotein Cholesterol; Hb: Hemoglobin; LDH: Lactate Dehydrogenase; SIRS: Systemic Inflammatory Response Syndrome; BISAP: Bedside Index for Severity in Acute Pancreatitis; APACHE II: Acute Physiology and Chronic Health Evaluation II; NPS: Naples Prognostic Score;. GI: Gastrointestinal; ICU: Intensive Care Unit; Column headers are bold for visual distinction. Bold text indicates variable category headers. Bold *p*-values indicate statistically significant differences (*p* < 0.05).

**Table 3:** Univariable and multivariable logistic regression analysis of predictors of severe acute pancreatitis.

Variable	*N*	Univariable Analysis				Multivariable Analysis			
		OR	95% CI Lower	95% CI Upper	*p* Value	OR	95% CI Lower	95% CI Upper	*p* Value
Age	452	1.009	0.993	1.026	0.274	0.998	0.979	1.017	0.835
Creatinine (mg/dL)	452	2.802	1.548	5.074	**<0.001**	2.038	1.264	3.287	**0.003**
CRP (mg/dL)	452	1.006	1.003	1.009	**<0.001**	1.004	1.001	1.007	**0.004**
Glucose (mg/dL)	442	1.004	1.000	1.008	**0.032**	1.004	1.000	1.008	**0.048**
NPS (high)	452	6.639	3.237	13.617	**<0.001**	4.346	2.018	9.362	**<0.001**
SIRS score	305	1.77	1.16	2.72	**0.008**				
BISAP score	301	2.08	1.51	2.91	**<0.001**				
Ranson score	393	1.25	0.96	1.63	0.092				
APACHE II score	64	1.26	1.12	1.47	**<0.001**				

OR: Odds Ratio; CI: Confidence Interval; CRP: C-Reactive Protein; NPS: Naples Prognostic Score; SIRS: Systemic Inflammatory Response Syndrome; BISAP: Bedside Index for Severity in Acute Pancreatitis; APACHE II: Acute Physiology and Chronic Health Evaluation II. Statistically significant *p*-values (*p* < 0.05) are shown in bold. Column headers are bold for visual distinction. Bold *p*-values indicate statistically significant differences (*p* < 0.05).

## Data Availability

The datasets used and/or analysed during the current study are available from the corresponding author on reasonable request.

## Supplementary Materials

The following supporting information can be downloaded at: https://www.mdpi.com/article/10.3390/jcm15176659/s1, Table S1: Variance inflation factors (VIF) for covariates in the multivariable logistic regression model; Table S2: Comparison of discriminative performance (AUC) of NPS and established prognostic scores in patients with complete data for all scores (*n* = 60).

## Abbreviations

Acute pancreatitis: AP, Naples Prognostic Score: NPS, Mild acute pancreatitis: MAP, Moderately severe acute pancreatitis: MSAP, Severe acute pancreatitis: SAP, Ranson score: RS, Acute Physiology and Chronic Health Evaluation II: APACHE II, Bedside Index for Severity in Acute Pancreatitis: BISAP, Systemic Inflammatory Response Syndrome: SIRS, NLR: neutrophil-to-lymphocyte ratio, LMR: lymphocyte-to-monocyte ratio.
