# Supplementary material for: Predictive Value of Naples Prognostic Score in Determining the Prognosis of Acute Pancreatitis—A Single-Center Experience
Source: J Clin Med. 2026 Aug 28;15(17):6659. doi: 10.3390/jcm15176659 (PMC13566422; doi:10.3390/jcm15176659)
Supplement: Supplementary file 1 [file jcm-15-06659-s001.zip › jcm-4461719-supplementary.pdf]

Supplementary Table S1. Variance inflation factors (VIF) for covariates in the multivariable logistic regression model

Covariate	Tolerance	VIF	Interpretation
Age	0.955	1.047	No multicollinearity
Creatinine (mg/dL)	0.943	1.061	No multicollinearity
CRP (mg/dL)	0.906	1.104	No multicollinearity
Glucose (mg/dL)	0.992	1.008	No multicollinearity
NPS (high vs. low)	0.859	1.164	No multicollinearity

Variance inflation factors (VIF) for covariates included in the multivariable logistic regression model (n = 442 complete cases), calculated using the car package in R. All VIF values were below the threshold of 5, indicating no evidence of significant multicollinearity among covariates. VIF: variance inflation factor; CRP: C-reactive protein; NPS: Naples Prognostic Score.

Supplementary Table S2. Comparison of discriminative performance (AUC) of NPS and established prognostic scores in patients with complete data for all scores (n = 60)

Score	n	Sensitivity	Specificity	AUC (95% CI)	p vs. NPS (DeLong) raw / adjusted*
SIRS	60	0.850	0.400	0.662 (0.521–0.787)	0.031 / 0.313
BISAP	60	0.550	0.700	0.692 (0.556–0.816)	0.048 / 0.384
Ranson	60	0.750	0.525	0.657 (0.511–0.795)	0.057 / 0.402
APACHE II	60	0.800	0.650	0.817 (0.696–0.918)	0.762 / 1.000
**NPS (ordinal, 0–4)**	**60**	**0.500**	**0.975**	**0.835 (0.723–0.928)**	**reference**

*Raw (unadjusted) and multiplicity-adjusted p-values from pairwise DeLong tests comparing each score's AUC with that of NPS. Prevalence of SAP in this subgroup was 33.3% (20/60), substantially higher than in the overall cohort (11.5%), as this subgroup was restricted to patients in whom APACHE II — which requires 12 physiological and laboratory variables typically available only in intensive care settings — could be calculated. NPS was modeled using its original five-level ordinal score (0–4), consistent with the primary analysis; the optimal cutoff in this subgroup (Youden method) was 3.5, closely approximating the cutoff of ≥3 identified in the full cohort. AUC: area under the receiver operating characteristic curve; SIRS: Systemic Inflammatory Response Syndrome; BISAP: Bedside Index for Severity in Acute Pancreatitis; APACHE II: Acute Physiology and Chronic Health Evaluation II; NPS: Naples Prognostic Score.
